# A systematic review and meta-analysis of the effect and safety of ginger in the treatment of pregnancy-associated nausea and vomiting

**DOI:** 10.1186/1475-2891-13-20

**Published:** 2014-03-19

**Authors:** Estelle Viljoen, Janicke Visser, Nelene Koen, Alfred Musekiwa

**Affiliations:** 1 Division of Human Nutrition, Faculty of Medicine and Health Sciences, Stellenbosch University and Tygerberg Academic Hospital, Cape Town, South Africa; 2 Centre for Evidence Based Health Care, Faculty of Medicine and Health Sciences, Stellenbosch University, Cape Town, South Africa; 3 Current affiliation: Discipline of Human Nutrition and Dietetics, School of Health Care Sciences, University of Limpopo, MEDUNSA Campus, Garankuwa, South Africa

**Keywords:** Pregnancy, Ginger, Nausea, Vomiting, Systematic review

## Abstract

**Background and objectives:**

Nausea and vomiting during pregnancy (NVP) occur commonly. Possible harmful side-effects of conventional medicine to the fetus create the need for alternative options to relieve NVP. This systematic review (SR) investigated current evidence regarding orally administered ginger for the treatment of NVP. The primary objective was to assess the effectiveness of ginger in treating NVP. The secondary objective was to assess the safety of ginger during pregnancy.

**Methods:**

A comprehensive electronic bibliographic database search was carried out. Randomized controlled trials (RCTs) of the efficacy of orally administered ginger, as treatment for NVP in pregnant women at any stage of pregnancy, published in English, were included. Two researchers independently extracted data and assessed trial quality. RevMan5 software (Cochrane Collaboration) was used for data analysis. p < 0.05 was considered statistically significant.

**Results:**

Twelve RCTs involving 1278 pregnant women were included. Ginger significantly improved the symptoms of nausea when compared to placebo (MD 1.20, 95% CI 0.56-1.84, p = 0.0002, I^2^ = 0%). Ginger did not significantly reduce the number of vomiting episodes during NVP, when compared to placebo, although there was a trend towards improvement (MD 0.72, 95% CI -0.03-1.46, p = 0.06, I^2^ = 71%). Subgroup analyses seemed to favor the lower daily dosage of <1500 mg ginger for nausea relief. Ginger did not pose a significant risk for spontaneous abortion compared to placebo (RR 3.14, 95% CI 0.65-15.11, p = 0.15; I^2^ = 0%), or to vitamin B_6_ (RR 0.49, 95% CI 0.17-1.42, p = 0.19, I^2^ = 40%). Similarly, ginger did not pose a significant risk for the side-effects of heartburn or drowsiness.

**Conclusions:**

This review suggests potential benefits of ginger in reducing nausea symptoms in pregnancy (bearing in mind the limited number of studies, variable outcome reporting and low quality of evidence). Ginger did not significantly affect vomiting episodes, nor pose a risk for side-effects or adverse events during pregnancy. Based on evidence from this SR, ginger could be considered a harmless and possibly effective alternative option for women suffering from NVP.

International Prospective Register of Systematic Reviews (PROSPERO) registration number: CRD42011001237.

## Introduction & rationale for the review

Nausea and vomiting are very common complaints during the early weeks of pregnancy. Due to the possible harmful side-effects that conventional medicine may pose to the unborn fetus, many mothers choose not to use it, and are left helpless against this burden. Nausea and vomiting of pregnancy (NVP) is commonly referred to as morning sickness (although it can occur at any time of the day or night), and affects about 80-90% of pregnant women in varying degrees [1,2]. Most of these women will experience both nausea and vomiting, and some only nausea without vomiting or retching, but vomiting alone is rare [2]. Symptoms usually appear at 4–9 weeks of gestation, reaching a peak at 7–12 weeks, and subsiding by week 16. About 15-30% of pregnant women’s symptoms will persist beyond 20 weeks, or even up to the time of delivery [1,2]. Hyperemesis gravidarum (HG) is severe and persistent vomiting during pregnancy, which can lead to dehydration, electrolyte disturbances and liver damage, possible fetal damage and in extreme cases, the death of the mother [1,3-5]. Women with HG usually need to be hospitalized [1] and it occurs in approximately 2% of pregnancies [1,2].

The exact cause of NVP remains unclear, and is probably multifactorial. Theories include the rapid increase in hormones such as estrogen and human chorionic gonadotropin (hCG), [6] or *Helicobacter pylori* (*H.pylori*) infection, as well as psychological and genetic predisposition [2,6]. Severe NVP and HG can lead to maternal malnourishment and weight loss, leading to negative fetal outcomes including low birth weight and preterm birth [1]. Maternal complications include acute renal failure, esophageal rupture, coagulopathy and on rare occasions, Wernicke’s encephalopathy [2]. The negative effects of NVP described clearly show the importance of managing and treating NVP and HG as early as possible, and not considering NVP as merely an unpleasant part of pregnancy that has to be endured and suffered through.

Pharmacological treatment of NVP is complicated due to the fact that during pregnancy, many physiological changes occur, including gastro-intestinal motility, plasma volume and glomerular filtration [7]. These factors all influence the distribution, absorption and excretion of drugs and due to this reason, not all drugs are safe during pregnancy. Many drugs cross the placenta by simple diffusion and can affect the fetus directly [7]. Non-pharmacological treatment of NVP includes ginger and simple lifestyle changes that have been described in the literature [1]. Acupressure is also a safe and non-invasive treatment for NVP, although there is a lack of evidence of efficacy [1,6].

Ginger (*Zingiber officinale* Roscoe) is widely used, with the most common ailments currently being treated with ginger including nausea, vomiting, pregnancy-associated morning sickness, motion sickness and indigestion [8-12]. There is mixed scientific evidence for the use of ginger in NVP [8,10]. It should be noted that high doses of concentrated ginger in the form of powder or herbal tinctures can increase bleeding risk by decreasing platelet-aggregation, and also increase stomach acid production, especially if taken with other herbs or medicines with the same effect [8,9,13]. Thus, ginger supplementation can have additive or competitive interactions with some medicines.

Several studies have been performed on the use of ginger as an anti-emetic for use with post-operative nausea and vomiting, motion sickness and vertigo and chemotherapy-induced nausea and vomiting [8,9,11,14]. The ingestion of oral ginger in a fasting state or after food intake results in an increase in gastro-duodenal motility [15], which could be a possible mechanism of action for the reduction in nausea and vomiting.

Currently no clear guidelines are available for ginger’s use in the treatment of NVP, despite some literature available on the subject [10,16,17]. A systematic review of the available literature (also focusing on safety aspects) can provide the best current evidence regarding possible benefits or risks for the clinical use of ginger to treat NVP.

### Objectives

The primary objective of this systematic review (SR) was to assess the effectiveness of ginger in the treatment of NVP. The secondary objective was to assess the safety of orally administered ginger in the treatment of NVP, by identifying adverse events or side-effects (if any), and to classify them as major (serious complications detrimental to the mother or fetus), or minor (discomfort, but manageable side-effects).

## Methods

### Ethics and protocol registration

As this SR utilizes data available in the public domain, it was exempt from ethical review by the Health Research Ethics Committee at Stellenbosch University (N11/04/127). The protocol was registered on the PROSPERO Register and can be viewed at http://www.crd.york.ac.uk/prospero/. Registration number CRD42011001237.

### Criteria for considering studies for this review

#### *Types of studies and participants*

Randomized controlled trials (RCTs) involving human participants, and investigating ginger for the treatment of NVP were included in this SR. Only studies that were published in English were included. Trials were included despite lack of blinding or placebo treatment. Women suffering from NVP were included, with no restriction on their age or stage of pregnancy (as included in the various trials).

#### *Types of interventions*

Any form of orally administered ginger intervention (fresh root, dried root, powder, tablets, capsules, liquid extract, and tea) compared with an inert (placebo) or active ingredient, was included.

#### *Types of outcome measures*

• Symptom scores on the subjective feeling of nausea, measured by standardized scales or methods [e.g. Visual Analogue Scale (VAS)].

• The incidence of vomiting episodes, measured by daily recording.

• The general response to the treatment, measured by standardized scales or methods (e.g. the 5-point Likert-type scale).

• The occurrence of adverse events and side-effects.

### Search methods for identification of studies

Literature searches were conducted in computerized databases from 1966 until 12 July 2013, with the help of a qualified medical librarian. Databases searched included Medline (accessed via Pubmed); EBSCO host, including Academic Search Premier, CINAHL (nursing & allied health research database), and CAB abstracts; CENTRAL (Cochrane Central Register of Controlled Trials); Science Direct; ISI Web of Science, ISAP (Index to South African Periodicals – National Library of South Africa); Proquest; Scopus Abstracts; Africa Wide; SABINET (South African Bibliographic Information Network); Current Controlled Trials (http://www.controlled-trials.com) and Clinical trials.gov (http://www.clinicaltrials.gov). The author team also searched for additional studies by searching the reference lists of the included trials and other articles identified by the electronic search. The final complete search word string was: Pregnan* AND (nausea OR vomit* OR morning sickness OR hyperemesis gravidarum) AND (ginger OR zingiber officinale roscoe) AND (clinical trial* OR randomized control trial* OR random allocation OR placebo* OR random research OR comparative OR “evaluation stud*” OR follow up OR prospective* OR control* OR volunteer* OR single mask* OR double mask* OR treble mask* OR tripl* mask* OR single-blind OR double-blind OR treble blind OR tripl* blind*).

### Selection of studies

Two reviewers independently assessed titles and abstracts of references retrieved from the searches and selected all potentially relevant studies. These potentially relevant articles were retrieved as full text in hard copy and assessed independently by the reviewers against the eligibility criteria, as described earlier. Disagreements were resolved with discussion and consensus. Studies that initially appeared to be relevant but were subsequently excluded were listed in a table of excluded studies with reasons for their exclusion.

### Data extraction and management

Data extraction was carried out in duplicate, independently, and differences were resolved by discussion and consensus. Data extraction forms were designed to tabulate the characteristics of the included studies. Where missing information was detected or clarity was needed, the authors of the primary studies were contacted via e-mail. Variables for which data were sought included study design, treatment and comparator, total number of participants at beginning and end of trial in intervention and control groups, length of treatment in days, outcomes, main results and adverse events reported.

### Assessment of quality of evidence

Two reviewers independently assessed the components of each trial for risk of bias, at study level. The Cochrane “risk of bias” assessment tool [18] was used to assess the potential sources of bias in the methodology of the included trials. The domains assessed were sequence generation, allocation concealment, blinding, incomplete outcome data, selective outcome reporting, and other potential threats to validity. Assessment was done by answering a pre-specified question about the adequacy of each individual study in relation to the entry, in such a way that the judgment of ‘yes’ was indicative of low risk of bias, ‘no’ was indicative of high risk of bias, and ‘unclear’ was indicative of uncertain risk of bias. Disagreements were resolved with discussion and consensus.

### Measures of treatment effect and data synthesis

Dichotomous outcomes (including adverse events, nausea and vomiting) were expressed as risk ratios (RR) with 95% confidence intervals (CI). Continuous outcomes such as symptom scores (for example, as measured by a VAS), were expressed as mean differences (MD) with 95% CI’s. Heterogeneity was assessed by both the visual inspection of the forest plots (where non-overlapping of confidence intervals indicated the likelihood of heterogeneity) and by using the Chi^2^-test for heterogeneity (differences at the level of p < 0.05 were considered to be statistically significant). Heterogeneity was also expressed as the I^2^ statistic, [18] with a value of 0% indicating no heterogeneity. The investigators undertook to assess funnel plots to explore the possibility of small study and other bias where at least ten studies were included per meta-analysis [18].

The Review Manager 5.0 (RevMan 5) computer program (developed by the Cochrane Collaboration) was used for data entry and statistical analysis of the data. A random effects model of meta-analysis was used in the presence of moderate heterogeneity of treatment effects, and a fixed effect model in the absence of heterogeneity. The Mantel-Haenszel (M-H) method of meta-analysis was used for dichotomous outcomes and the Inverse-Variance (IV) method was used for continuous outcomes. All statistical methods used were confirmed by a statistician trained in meta-analyses and systematic reviews.

### Subgroup and sensitivity analyses

The planned subgroup analyses to explore possible sources of heterogeneity included different dosages administered in the various studies [low (<1500 mg ginger/day) vs. high (≥ 1500 mg ginger/day)] and different durations of intervention in the various studies [short treatment (<7 days) vs. long treatment (≥7 days)]. Sensitivity analyses were planned to explore the influence of study quality and source of funding on effect size, should sufficient studies exist.

## Results

### Study selection

**Figure 1:**
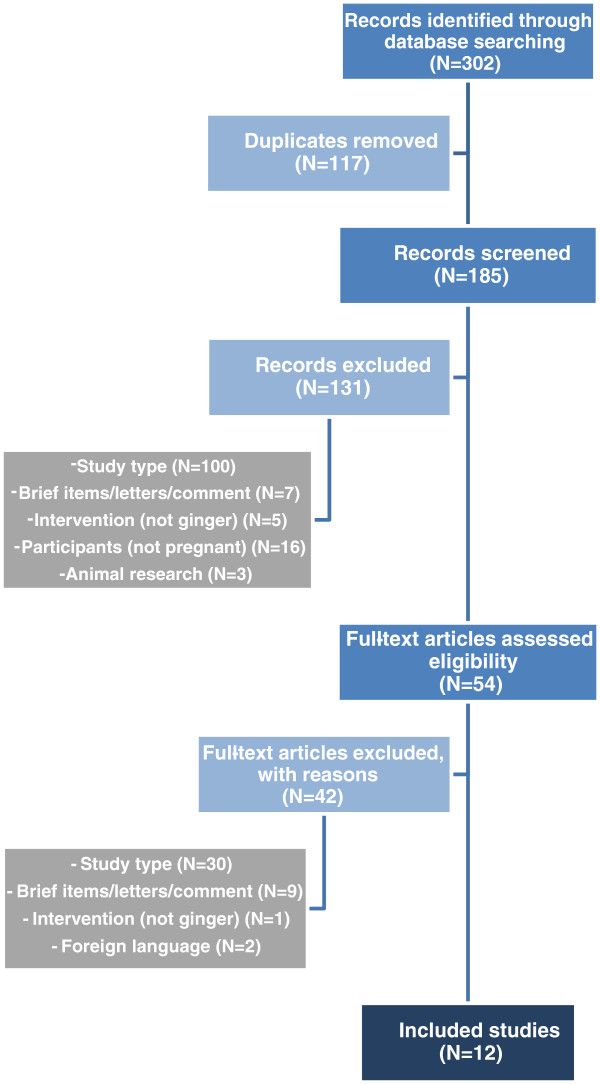
Diagrammatic representation of the process followed in the selection of studies.

The process followed in the selection of studies and the results obtained from the search are shown in Figure 1. Across all searched databases (and reference lists reviewed) 302 abstracts were identified as potentially relevant. Of these, 117 studies were identified as duplicates. In the case of duplicate publications, the original paper (or the oldest version) was used. A further 173 were excluded, in 2 phases, for various reasons (Figure 1). Finally, twelve studies [19-30] met the aforementioned criteria and were included. Although foreign language studies were excluded from this review, all potentially eligible studies reported in languages other than English were documented for future assessment [31,32].

### Study characteristics

Included studies were published from 1991 to 2011. Key characteristics of the included studies are presented in Table 1 and arranged alphabetically. Eleven (91.6%) of the twelve included trials were designed as parallel group studies. Only one study [22] had a cross-over design. A total of 1278 participants were included in the respective studies, ranging from 26 in the smallest trial [23] to 291 participants in the largest trial [27]. Only 2 trials [26,27] recruited more than 150 participants. Eleven of the twelve included studies [19-21,23-30] (91.6%), included women suffering from NVP, and one study [22] (8.3%) included women suffering from HG. The study intervention (ginger) was clearly described in each included study. Most of the studies (n = 8) (66.7%) [20-22,25-29] used ginger powder capsules as intervention, ranging from 1000 mg to 1950 mg ginger per day. One study [19] (8.3%) used ginger biscuits as intervention, with a total dose of 2500 mg ginger per day. One study [23] (8.3%) used a total of 1000 mg ginger syrup per day, dissolved in water; one study [30] (8.3%) used 1000 mg ginger extract per day, in capsule form; and one study [24] used a total of 600 mg ginger essence per day. The comparator was clearly described in most of the included studies [20-23,25-28,30]. A placebo was used as the control in 7 studies. Two studies [22,25] used lactose as the placebo, one used lemon oil [23], one used flour [24], and one used soy bean oil [30]. One study [19] used placebo biscuits but did not specify the content of the biscuit, and one study [29] did not specify the content of the placebo capsule. Four studies used Vitamin B_6_ as active comparator. Two studies [20,27] used 70 mg per day, one [21] used 40 mg per day and one [28] used 30 mg Vitamin B_6_ per day. One study [24] used 30 mg Metoclopramide as comparator, as well as placebo. The remaining study [26] used 100 mg Dimenhydrinate per day as active comparator (Table 1).

**Table 1:** Characteristics of included studies

**Study ID**	**Risk of bias**	**NPB/NME (treatment)**	**Intervention (ginger dose/day)**	Comparator* (dose per day)	**L of T (days)**	**Main outcome measures**	**Main results**
**Basirat [**[19]**]**	High	65/62 (32G, 30C)	Ginger biscuits (500 mg 5 times daily = 2500 mg/day)	Placebo biscuit (5 biscuits per day, dose not specified)	4	Severity of nausea (VAS 0–10); number of vomiting episodes; general response to treatment (5-item Likert scale)	Ginger biscuits provided significantly greater relief from the severity of nausea (p = 0.01), and to some extent vomiting (p = 0.24).
**Chittumma [**[20]**]**	High	126/123 (61G, 62C)	Ginger powder capsules (325 mg ×2, three times daily, = 1950 mg/day)	Vitamin B_6_ capsules (12.5 mg ×2, three times daily =75 mg/day)	4	Change in nausea and vomiting scores (3 symptoms on Rhodes index); occurrence of side-effects	Results showed that ginger is significantly more effective in relieving NVP than vitamin B6 (p < 0.05).
**Ensiyeh [**[21]**]**	High	70/69 (35G, 34C)	Ginger powder capsules (500 mg 2×/d =1000 mg/day)	Vitamin B_6_ capsules (20 mg twice per day =40 mg/day)	4	Severity of nausea (VAS 0–10); number of vomiting episodes; general response to treatment (5-item Likert scale); occurrence of side-effects or adverse pregnancy outcome	The results showed that the ginger is significantly more effective than vitamin B6 for relieving the severity of nausea (p < 0.024), and equally effective for reducing the number of vomiting episodes.
**Fischer-Rassmussen [**[22]**]**	Mode-rate	30/27 (27G, 27C) (cross-over**)	Ginger powder capsules (250 mg 4 times per day = 1000 mg/day)	Placebo capsules (lactose) (250 mg 4 times per day = 1000 mg/day)	4	Preference of treatment period; relief scores (4-point scoring system); outcome of pregnancy	The results showed that ginger was significantly more effective than the placebo in eliminating or minimizing HG (p = 0.035).
**Keating [**[23]**]**	High	26/21 (12G, 9C)	Ginger syrup in water (250 mg 4 times per day = 1000 mg/day)	Placebo syrup (lemon oil) 4x/day (dose not specified)	14	Level of nausea (numerical scale 1–10); number of vomiting episodes	Ginger had a greater effect on the relieving of NVP, but due to the small study sample the results were not statistically analyzed. The authors concluded that ginger syrup may be more effective than placebo syrup in treatment of NVP.
**Mohammadbeigi [**[24]**]**	High	102/102 (34G, 34C1, 34C2)	Ginger essence capsules (200 mg 3×/day = 600 mg/day)	**1**. Metoclopramide capsules (10 mg 3×/day = 30 mg/day)	5	Used RINVR to measure severity of nausea and vomiting.	Ginger was less effective than metoclopramide in reducing nausea and vomiting during pregnancy, but the difference was not statistically significant (p = 0.509).
				**2**. Placebo capsules (flour) (200 mg 3×/day = 600 mg/day)			
**Ozgoli [**[25]**]**	Mode-rate	70/67 (32G, 53C)	Ginger powder capsules (250 mg 4 times per day = 1000 mg/day)	Placebo capsules (lactose) (250 mg 4 ×/d 1000 mg/day)	4	Nausea intensity (VAS 0–10); number of vomiting incidences	The results showed that ginger was significantly more effective than the placebo in improving symptoms of NVP (p < 0.05).
**Pongrojpaw [**[26]**]**	High	170/151 (77G, 74C)	Ginger powder capsules (500 mg 2x/d =1000 mg/day)	Dimenhydrinate capsules (50 mg 2x/d = 100 mg/day)	7	Degree of nausea (VAS 0–10); number of vomiting incidences; occurrence of side-effects	There was no significant difference in the visual analogue nausea scores between the two groups. Ginger was as effective as dimenhydrinate in the treatment of NVP, and has fewer side-effects.
**Smith [**[27]**]**	High	291/235 (120G, 115C)	Ginger capsules (350 mg 3times per day = 1050 mg/day)	Vitamin B_6_ capsules (25 mg 3x/d =75 mg/day)	21	Nausea, vomiting and dry retching on days 0,7,14,21 (Rhodes Index of Nausea and Vomiting Form2) (5-point Likert scale); change in health status on day 0,21 (MOS 36 Short Form Health Survey, 8-multi-item scale, higher core = better outcome); occurrence of side-effects and adverse pregnancy outcomes	The results indicated that ginger is equivalent to vitamin B6 in improving nausea, dry retching and vomiting in pregnancy. All p-values were <0.001.
**Sripramote [**[28]**]**	High	138/128 (64G, 64C)	Ginger powder capsules (500 mg 3×/d 1500 mg/day)	Vitamin B_6_ capsules (10 mg 3×/d =30 mg/day)	3	Severity of nausea (VAS 0–10); number of vomiting incidences; occurrence of side-effects	Both ginger and vitamin B6 were effective for treating NVP (p < 0.001). There were no significant differences between the two treatments’ efficacy.
**Vutyavanich [**[29]**]**	High	70/67 (32G, 35C)	Ginger powder capsules (250 mg 4x/day =1000 mg/day)	Placebo capsules (not specified) (250 mg 4x/day = 1000 mg/day)	4	Severity of nausea (VAS 0–10); number of vomiting episodes; general response to treatment after 1 week (5-item Likert scale); occurrence of side-effects and adverse pregnancy outcomes	Ginger was significantly more effective than the placebo in relieving the severity of nausea in pregnancy (p = 0.014).
**Willetts [**[30]**]**	Mode-rate	120/99 (48G, 51C)	Ginger extract capsules (125 mg 4x/d =1000 mg/day)	Placebo capsules (soy bean oil 4x/d) (dose not specified)	4	Used RINVR to measure frequency, duration, distress caused by nausea, vomiting and retching; long term follow-up for birth outcome	Ginger was more effective than placebo for improving nausea and retching during pregnancy, but no difference in the vomiting episodes were observed. No p-values were provided.

*Comparator: includes placebo and active ingredients. **Cross-over design RCT. All the other studies were parallel design RCT’s.

**RCT**: Randomized controlled trial; **NVP**: Nausea and vomiting of pregnancy; **HG**: Hyperemesis gravidarum; **NPB**: Number of patients at beginning of trial; **NPE**: Number of patients at end of trial; **L of T**: Length of treatment; **G**: patients in Ginger group; **C**: patients in comparator group **C1**: control group nr 1; **C2**: control group nr 2; **VAS**: Visual analogue scale; **MOS**: Medical outcome study; **RINVR**: Rhodes Index of Nausea, Vomiting and Retching. 5-point Likert type tool with 8 items.

### Characteristics of outcome measures

Nausea (the feeling of being about to vomit) is a subjective feeling, and several tools have been developed to reliably measure it. In contrast to nausea, vomiting is a readily observable occurrence that can be measured or reported without information from the patient. Still, the distress caused by vomiting cannot be observed by another person, and remains a subjective feeling. The included studies used a variety of tools to measure nausea severity and vomiting incidences. Six of the studies [19,21,25,26,28,29] used a visual analogue scale (VAS) of 0–10 centimeters to score nausea severity, and participants recorded the number of vomiting episodes daily. One study [23] used a numerical scale of 1–10 for scoring nausea severity, as well as daily recording of vomiting episodes. Four studies [20,24,27,30], used the Rhodes Index of Nausea and Vomiting, or parts of this index, to measure both nausea and vomiting, and the remaining study [22] used a 4 point system to score nausea and vomiting symptoms.

#### *General response to treatment*

The tools used in the included studies to measure the general response to treatment outcome were Likert scales [19,21,29], a point-system instrument [22] and the Medical outcome survey (MOS) Short form-36 Health Survey [27,33].

#### *Adverse events and side-effects*

The judgments made on the seriousness of the reported side-effects or adverse events are the author team’s own subjective judgments, also taking into account the fact that some of these events can occur in a normal pregnancy, without any interventions. Adverse events and side effects were classified as major when it was considered a serious complication, possibly being detrimental to the mother or fetus. Major events reported across the studies were allergic reaction [30], arrhythmia [20], dehydration [30], and spontaneous abortion [21,22,29]. Events were classified as minor when considered a discomfort, but manageable side-effects. Minor events reported across the studies included abdominal discomfort [29], belching [27], burning sensation after capsule ingestion [27], diarrhea [29], dry retching or vomiting after capsule ingestion [27], headaches [20,29], drowsiness [19,20,26,28] and heartburn [19,20,26,28-30].

### Methodological quality

**Figure 2:**
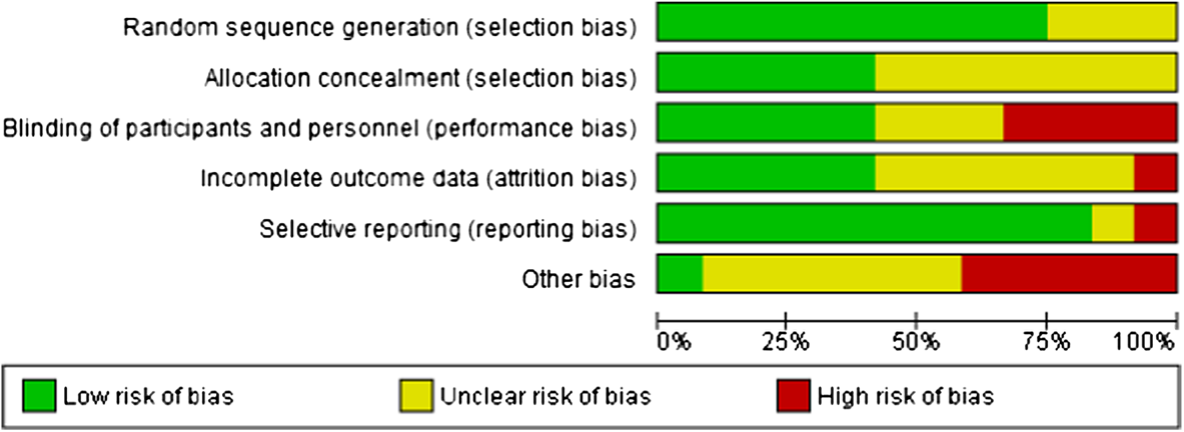
Methodological quality graph: judgments about each methodological quality item presented as percentages for all included studies (n = 12).

All included trials were RCT’s. The Cochrane “risk of bias” assessment tool [18] was completed for each of the included studies to assess methodological quality and to enable data entry into the RevMan 5 program. The author team’s judgments about each methodological quality assessment factor across all included studies are demonstrated in Figure 2, and indicate high risk of bias in especially the “blinding” and “other bias” categories. All included studies were concluded to be either at high [19-21,23,24,26-29] or moderate [22,25,30] risk of bias.

Only one [19] of the twelve studies (8.3%) had no risk of other bias. Six [22-25,27,30] of the studies (50%) had an unclear risk of other bias, and five [19,21,26,28,29] studies (41.6%) had a high risk of other bias. The high risk studies all included dietary counseling as part of their treatment in both the experimental and the control groups. The authors considered this as a possible confounding factor, since change in outcome scores could be affected by the dietary adjustments made, rather than the intervention itself. No reporting was done on the dietary measures in any of these mentioned studies.

### Effect of interventions

The included studies were split into four groups, according to the comparison substance used. Placebo was used in seven studies (considered a control substance). Four studies used Vitamin B_6_, one study used Dimenhydrinate, and one study used Metoclopramide as comparator (these three substances were considered active ingredients, and not controls). The Metoclopramide study compared ginger to both Metoclopramide and placebo.

The analyses for the different active ingredients were done separately as these were different comparisons and they could not be pooled in one meta-analysis. Subgroup analyses addressing dosage and duration aspects were performed for the primary objectives, namely the effectiveness of ginger for reduction in nausea and vomiting. No sensitivity analyses were performed as a result of an insufficient number of studies per comparison group. There were insufficient studies per comparison and outcome to permit the use of funnel plots to assess publication bias.

### Comparison 1: Ginger versus Placebo

Seven studies assessed the effect of ginger versus placebo [19,22-25,29,30].

#### *Improvement in nausea symptoms*

**Figure 3:**
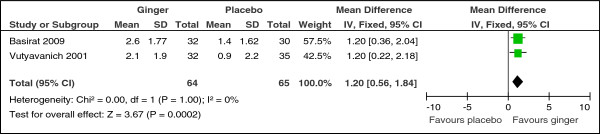
Forest plot of the improvement in nausea symptoms measured by change in VAS scores (ginger versus placebo).

All seven studies assessing the effect of ginger versus placebo reported this outcome but their results could not all be pooled in a meta-analysis. Two studies [19,29] reported the reduction in the visual analogue scale of post-therapy minus baseline nausea as mean and standard deviation (SD) and results were pooled in a meta-analysis. Ginger significantly decreased nausea symptoms when compared to placebo (MD 1.20, 95% CI: 0.56 to 1.84, p = 0.0002) (Figure 3) and there was no significant heterogeneity detected between the two studies (Chi^2^ = 0.00, p = 1.00, I^2^ = 0%). There were no significant subgroup differences between the higher dose (≥1500 mg daily) and the lower dose (<1500 mg daily) with respect to the improvement in nausea symptoms (change in VAS scores) (Chi^2^ = 0.00, p = 1.00, I^2^ = 0%). No subgroup analysis with respect to duration was undertaken, as the two studies had the same duration of 4 days.

One study [24] reported improvement in nausea severity using a Rhodes Index questionnaire during days 1–5. The observed trend showed a significant reduction in nausea severity in favor of ginger compared to the placebo group (p = 0.003) at the second to fifth day of treatment compared to the first day.

Two studies [23,25] reported this outcome in terms of the number of women showing improvement in nausea symptoms (again measured by VAS scores). Meta-analysis of the results from these two studies shows that ginger failed to decrease nausea symptoms when compared to the placebo (RR 2.00, 95% CI: 0.77 to 5.19, p = 0.15) and there may be moderate heterogeneity between the two studies (Chi^2^ = 2.42, p = 0.12, I^2^ = 59%). There were no subgroup differences between the longer duration (≥7 days) and the shorter duration (<7 days) with respect to the improvement in nausea symptoms (number showing significant improvement) (Chi^2^ = 2.04, p = 0.15, I^2^ = 50.9%). No subgroup analysis with respect to dose was undertaken, as the two studies had the same dosage of 1000 mg/day.

One study [30] reported the trend in mean nausea experience scores for both the ginger and placebo groups in the form of a figure only, from which no mean and SD values could be extracted. No treatment effect could therefore be calculated.

The remaining study [22] was a crossover study which reported the relief scores on symptoms of a combination of nausea, vomiting, change in body weight, and patient’s opinion about the treatment. The observed values for the relief scores were reported for each patient during the two periods of the crossover study in the form of a table. These values were used in calculating the mean difference (MD) and its standard error (SE) using a paired analysis and the 95% CIs were calculated using the generic-inverse variance method in RevMan 5. A significantly greater relief of the symptoms was found after ginger treatment compared to the placebo (MD 3.52, 95% CI: 0.27 to 6.77).

#### *Reduction in the number of vomiting episodes*

All seven studies in this comparison reported a reduction in the number of vomiting episodes, but not all their results could be pooled in a meta-analysis. Two studies [19,29] reported this outcome in the form of mean and SD, and their results could be pooled in a meta-analysis. According to the meta-analysis, ginger failed to significantly reduce the number of vomiting episodes compared to the placebo, although it did approach significance (MD 0.72, 95% CI: -0.03 to 1.46, p = 0.06) and statistically significant heterogeneity was detected between the two studies (Chi^2^ = 3.44, p = 0.06, I^2^ = 71%). There were no significant subgroup differences between the higher dose (≥1500 mg daily) and the lower dose (<1500 mg daily) with respect to the reduction in the number of vomiting episodes (Chi^2^ = 3.44, p = 0.06, I^2^ = 71%). No dose–response effect was found for this outcome.

One study [24] reported improvement in vomiting severity using a Rhodes Index questionnaire during days 1–5. The observed trend showed a significant reduction in vomiting severity in favor of ginger compared to the placebo group (p = 0.046) at the second to fifth day of treatment compared to the first day.

One study [22] reported vomiting in conjunction with nausea scores (as discussed in previous section). One study [23] reported the number of women who stopped vomiting by day 6 of treatment. Treatment with ginger failed to reduce the number of women who stopped vomiting by day 6, when compared to the placebo treatment (RR 3.33, 95% CI: 0.91 to 12.26). One study [25] reported that incidence of vomiting decreased by 50% in the ginger group and 9% in the placebo group, but this information is insufficient for calculation of a treatment effect. One study [30] only reported that there was no significant difference between ginger extract and placebo groups for any of the vomiting symptoms but failed to give any values for the calculation of a treatment effect, as mentioned earlier.

### General response to treatment

Only 3 of the 12 studies included in this SR reported on this outcome. One study [19] reported that ginger did not significantly result in better responses to the treatment, when compared to the placebo.

#### *The occurrence of adverse events and side effects*

Four studies [19,23,25,29] reported that none of the participants experienced any adverse events from ginger during the treatment period.

One study [22] reported that one patient had a spontaneous abortion and one patient asked for a legal abortion. Because this trial had a crossover design and all patients received both treatments, no treatment effect could be calculated for the occurrence of spontaneous abortion after the treatment period.

For all reported adverse events and side-effects in the various studies [including allergic reaction [30], dehydration [30], spontaneous abortions [29,30], abdominal discomfort [29], diarrhea [29], drowsiness [19], headache [29], heartburn [19,29,30], worsening of symptoms requiring pharmaceutical treatment [30] there were no significant differences between the ginger and placebo treated groups (Table 2).

**Table 2:** **Pooled estimates of effect size (95% confidence intervals) expressed as weighted relative risk for adverse events and side-effects of ginger versus control group (Placebo, Vitamin B**_**6**_**, Dimenhydrinate)**

**Outcome**	**Number of studies**	**RR**	**95% CI**	**Heterogeneity**	
				**Chi**^**2**^	**I**^**2 **^**(%)**
***Ginger versus placebo***					
^#^Allergic reaction [30]	1	3.00	0.12 to 72.20		
^#^Dehydration [30]	1	3.00	0.12 to 72.20		
^#^Spontaneous abortions [29,30]	2	3.14	0.65 to 15.11	0.00	0
Abdominal discomfort [29]	1	3.27	0.14 to 77.57		
Diarrhea [29]	1	3.27	0.14 to 77.57		
Drowsiness [29]	1	2.82	0.12 to 66.62		
Headache [29]	1	1.31	0.44 to 3.89		
Heartburn[19,29,30]	3	5.03	0.89 to 28.61	0.35	0
Worsening of symptoms requiring pharmaceutical treatment [30]	1	0.33	0.01 to 8.02		
***Ginger versus vitamin B***_***6***_					
^#^Arrhythmia [20]	1	0.51	0.05 to 5.46		
^#^Spontaneous abortions [21,27]	2	0.49	0.17 to 1.42	1.67	40
Belching [27]	1	27.18	1.63 to 453.06*		
Burning sensation after capsule ingestion [27]	1	1.01	0.21 to 4.91		
Drowsiness [20,28]	2	0.75	0.48 to 1.19	0.18	0
Dry retching [27]	1	0.93	0.76 to 1.15		
Heartburn [20,28]	2	2.35	0.93 to 5.93	1.03	3
Vomiting [27]	1	1.51	0.26 to 8.91		
***Ginger versus Dimenhydrinate***					
Drowsiness [26]	1	0.08	0.03 to 0.18**		
Heartburn [26]	1	1.44	0.65 to 3.20		

^#^Major adverse events (serious complications, possibly detrimental to the mother or fetus) (authors’ judgement); rest considered minor (discomfort, but manageable side effects) - sorted alphabetically, first major then minor events.

*Indicates significant finding: Ginger significantly increased the risk of belching compared to vitamin B_6_.

**Indicates significant finding: Dimenhydrinate significantly increased the risk of drowsiness compared to ginger.

*RR*, Relative risk; *CI*, confidence interval.

### Comparison 2: Ginger versus Vitamin B_6_

Four of the included studies assessed the effect of ginger versus vitamin B_6_[20,21,27,28].

#### *Improvement in nausea symptoms*

**Figure 4:**
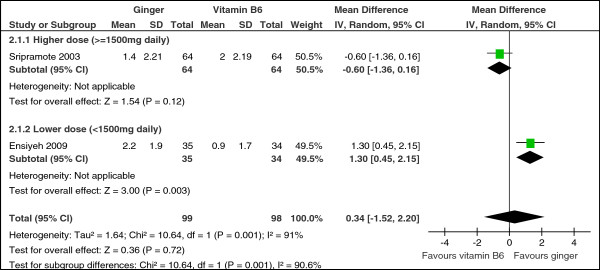
**Forest plot of the improvement in nausea symptoms as measured by the change in VAS scores (ginger versus Vitamin B6): subgroup analysis regarding dose (**≥**1500 mg versus < 1500 mg).**

All four studies assessing the effect of ginger versus vitamin B_6_ reported this outcome, but their results could not all be pooled in a meta-analysis. Two studies [21,28] reported the reduction in the VAS scores of post-therapy minus baseline nausea as mean and SD and their results were pooled in a meta-analysis. According to this meta-analysis, ginger failed to significantly decrease nausea symptoms when compared to vitamin B_6_ (MD 0.34, 95% CI: -1.52 to 2.20, p = 0.72) and significant heterogeneity was detected between the two studies (Chi^2^ = 10.64, p = 0.001, I^2^ = 91%). There were significant subgroup differences between the higher dose (≥1500 mg daily) and the lower dose (<1500 mg daily) with respect to the improvement in nausea symptoms (change in VAS scores) (Chi^2^ = 10.64, p = 0.001, I^2^ = 90.6%) (Figure 4). This implies a dose–response effect for this outcome in favour of the lower dosage. The different dosages between the two studies may be the source of heterogeneity detected in this meta-analysis.

No subgroup analysis with respect to duration was undertaken, as the two studies had the similar short durations of 4 days and 3 days.

One study [27] reported the reduction in nausea symptoms from baseline using the Rhodes Index of Nausea (ranging from 0 to 12, with larger scores indicating more symptoms). The results were reported in the form of mean and standard error (SE) and these values were used in calculating the SDs. The means and SDs were used in calculating the mean difference (MD) and its 95% CIs. There was no statistically significant improvement of nausea symptoms with ginger treatment compared to vitamin B_6_ treatment (MD -0.3, 95% CI: -0.85 to 0.25).

The remaining study [20] reported the reduction in nausea vomiting scales (episodes of nausea, duration of nausea, and number of vomits) using a modified Rhodes’ score. The results were reported in form of mean and SD and were used in calculating the MD which showed that ginger treatment significantly improved the nausea and vomiting symptoms compared to vitamin B_6_ treatment (MD 0.70, 95% CI: 0.20 to 1.20).

#### *Reduction in the number of vomiting episodes*

All four studies in this comparison group reported a reduction in the number of vomiting episodes, but not all the results could be pooled in a meta-analysis. Results from three studies [21,27,28] were reported in the form of mean and SD which were pooled in a meta-analysis. According to this meta-analysis, ginger failed to reduce the number of vomiting episodes when compared to vitamin B_6_ (MD -0.07, 95% CI: -0.48 to 0.35, p = 0.76) and there may have been moderate heterogeneity between the three studies (Chi^2^ = 3.58, p = 0.17, I^2^ = 44%).

There were no significant subgroup differences between the higher dose (>1500 mg daily) and the lower dose (<1500 mg daily) with respect to the reduction in the number of vomiting episodes (Chi^2^ = 0.72, p = 0.40, I^2^ = 0%). Similarly there were no significant subgroup differences between the longer duration (≥7 days) and the shorter duration (<7 days) with respect to the reduction in the number of vomiting episodes (Chi^2^ = 3.51, p = 0.06, I^2^ = 71.5%).

The remaining one study [20] reported vomiting in conjunction with nausea as mentioned above.

#### *General response to treatment*

A meta-analysis of two studies [21,27] showed that ginger did not significantly increase the number reporting improvement when compared to vitamin B_6_.

#### *The occurrence of adverse events and side-effects*

Two studies [20,28] reported that none of the participants experienced any adverse events from either ginger or vitamin B_6_ during the treatment period.

Ginger significantly increased the risk of belching compared to vitamin B_6_ (RR 27.18, 95% CI: 1.63 to 453.06) in one study [27]. For all other adverse events and side-effects reported in the various studies [including arrhythmia [20], spontaneous abortions [21,27], burning sensation after capsule ingestion [27], drowsiness [20,28], dry retching [27], heartburn [20,28], vomiting [27]] there were no significant differences between the ginger and Vitamin B_6_ treated groups (Table 2).

### Comparison 3: Ginger versus Dimenhydrinate

Only one included study [26] assessed the effect of ginger versus dimenhydrinate. This study reported a reduction in the VAS scores of post-therapy minus baseline nausea, as well as reduction in the number of vomiting episodes in the form of a figure only, from which no mean and SD values could be extracted. No treatment effect could therefore be calculated.

No adverse events were reported. The study reported results on drowsiness (minor side effect), with dimenhydrinate significantly increasing the risk of drowsiness compared to ginger (RR 0.08, 95% CI: 0.03 to 0.18). The study also reported results on heartburn (minor side effect) and there was no statistically significant difference found between the ginger and dimenhydrinate treated groups (Table 2).

### Comparison 4: Ginger versus Metoclopramide

Only one included study [24] assessed the effect of ginger versus metoclopramide.

#### *Improvement in nausea symptoms*

One study [24] reported improvement in nausea severity using a Rhodes Index questionnaire during days 1 to 5. There was no significant difference in the observed trend in nausea severity between the ginger and metoclopramide groups (p = 0.683) at the second to fifth day of treatment compared to the first day.

#### *Improvement in vomiting*

One study [24] reported improvement in vomiting severity using a Rhodes Index questionnaire during days 1 to 5. There was no significant difference in the observed trend in vomiting severity between the ginger and metoclopramide groups (p = 0.718) at the second to fifth day of treatment compared to the first day. No adverse events were reported in this study.

## Discussion

Literature indicate that the exact cause and treatment of NVP is still unclear [2,6,17,34-37]. Mothers and health practitioners often investigate alternative options to alleviate symptoms of NVP, due to the possible harmful side effects that conventional medicine may pose to the unborn fetus. In this regard, ginger is considered by many as a possible non-pharmacological treatment option for NVP. This updated systematic review has investigated the current evidence-base and supports and strengthen previous findings that ginger could be considered a harmless and possibly effective alternative option for women suffering from the symptoms of NVP.

### Primary outcomes

#### *Symptomatic relief of nausea, number of vomiting episodes and general response to treatment*

Ginger versus placebo was assessed in seven of the included studies [19,22-25,29,30]. Individually, all seven studies concluded that ginger was more effective than the placebo in relieving the intensity of nausea, or NVP in general. One meta-analysis of two studies [19,29] in this SR showed that ginger significantly decreased nausea symptoms when compared to placebo. When taking into account that other SRs [10,16,17,35,36] as well as the above-mentioned 7 concluded that ginger had beneficial effects on nausea during pregnancy (together with the findings of this SR), it is probably safe to assume that ginger has potential as a possible anti-emetic drug-alternative during pregnancy. The theoretical physiological mechanism by which ginger affects the digestive system also supports this theory. Ginger can increase gastric contractility, speeding up gastric emptying, and therefore increasing the gastro-intestinal transit time of meals, which can decrease the feeling of nausea [15].

Although three [22,25,29] of the seven studies that assessed ginger versus placebo concluded individually that ginger was more effective than placebo in reducing the number of vomiting episodes, the remaining evidence and meta-analysis performed lead to the conclusion that ginger did not significantly reduce the number of vomiting episodes during NVP when compared to the placebo. A meta-analysis of three studies [21,27,28] showed that ginger did not significantly reduce vomiting episodes when compared to vitamin B_6_. Based on this currently available evidence, the author team concludes that ginger does not seem to reduce the number of vomiting episodes significantly when compared to vitamin B_6_.

Due to the small number of studies reporting on the outcome of general response to treatment, no conclusions can be drawn in this regard.

### Secondary outcomes

#### *Adverse events and side-effects*

The author team made subjective judgments to classify the occurring adverse events and side-effects as major (serious complications detrimental to the mother or fetus, including arrhythmia, spontaneous abortion, allergic reaction to treatment, and dehydration), or minor (discomfort, but manageable side-effects). According to the available evidence (Table 2), ginger significantly increased the risk of belching compared to vitamin B_6_ (bearing in mind the very large CI indicating poor precision and thus limiting firm conclusions). Dimenhydrinate significantly increased the risk of drowsiness compared to ginger. Ginger therefore does not seem to pose a risk for any major side-effects or adverse events occurring, and thus no risk for any serious complications detrimental to the mother or fetus.

### Comparisons with other studies

The findings of this updated SR compare well with the findings of previously conducted reviews [10,14,16,17,34-36] on the same topic. Limited meta-analysis could be performed, often due to the heterogeneity in participants, interventions, outcome measures and comparison groups encountered. This clearly shows the need for more research on the topic, with larger studies and standardization of methods and materials. All these reviews suggest that ginger may be effective for the treatment of NVP, but data is insufficient to draw firm conclusions regarding the dosage and duration of treatment.

Two recently published systematic reviews on the same topic support the potential of ginger as a possible treatment option for NVP [35,36]. The SR by Ding et al. [35] included studies published from 2000–2009 and included 4 RCTs [23,25,27,30], all of which were also included in the current SR. This group reported that ginger was more effective than placebo, and as effective as Vitamin B_6_ in improving NVP. They concluded that ginger use for NVP were safe, but highlighted the need for further studies with longer duration, to establish the long term safety and effectiveness of ginger.

The SR by Thomson et al. [36] was published early in 2014 and included six studies [19,22,23,25,27,29], all of which were also included in the current SR. This meta-analysis indicated that ginger is better than placebo in improving NVP (RR 1.76, 95% CI: 1.18 to 2.65).

Both these recent systematic reviews used the approach of comparing ginger to placebo (both also including one study comparing ginger to Vitamin B_6_[27]). This current updated SR builds substantially on previous reviews by including a recent literature search and grouping all comparators into four groups (placebo, Vitamin B_6_, dimenhydrinate and metoclopramide), as any other treatment than placebo was considered an active ingredient.

### Practice points

From a practice point of view, the subgroup analysis performed indicated that the lower dosage of <1500 mg ginger per day could possibly be more effective than the higher dosage of ≥1500 mg (again, bearing in mind the limited value of the small subgroup analyses). Most studies provided 1000 mg of ginger powder for a period of 4 days to women suffering from NVP (with no apparent side-effects or adverse events). The literature suggests taking the total dose in three to four divided doses during the day, irrespective of mealtimes [21-23,25,26,29,30]. Mothers can be advised to use ginger freely in their cooking, to drink ginger tea and soft drinks, and to have dry ginger biscuits as needed.

### Strengths and limitations

This updated SR includes the latest trials related to the topic, with the last search for studies performed in July 2013. The small sample sizes and few study numbers analyzed per outcome, as well as differences in dosage and duration of treatment lead to high levels of inconsistency and heterogeneity in the results of the review. Unfortunately, many of the included studies did not present data in a usable form for inclusion in meta-analysis, or similar outcomes were reported differently and could not be pooled together. These factors limit the strength of evidence and cause some degree of uncertainty when interpreting the results. None of the twelve studies included in this SR described any form of chemical or chromatographic tests to verify the exact composition of the active compounds in the ginger preparations. Another limitation of this SR is the inclusion of only English language studies, although foreign language studies were documented for possible future inclusion.

As with many nutrition-related research studies, it is difficult to control every exposure and it is almost impossible to keep all dietary exposures identical for all participants. Ginger has a very characteristic and recognizable taste, which makes it difficult to mask during trials. This could act as a potential confounder, as it can be considered “unblinding” in some cases. A possible solution to this problem is to do pre-trial testing, as was done in the study by Vutyavanich et al., [29] to test if the patients are able to identify the treatment before the start of the trial. Publication bias is always a concern when a SR is conducted, as it is known that studies with negative results are often not published. Ginger is considered a complementary and/or alternative medicine (CAM) [37]. The publication of literature on CAM therapies might be suboptimal [10,37]. As with all SRs, there was potential for bias at all stages of the reviewing process. Minimizing bias was attempted by having two independent reviewers undertaking study selection, data extraction and quality assessment.

## Conclusion

This review suggests potential benefits of ginger in reducing nausea symptoms in pregnancy (bearing in mind the limited number of studies, variable outcome reporting and low quality of evidence). Subgroup analyses seemed to favor the lower daily dosage of <1500 mg ginger for nausea relief. Ginger did not have a significant impact on vomiting episodes, nor pose a risk for side-effects or adverse events during pregnancy. Based on evidence from this SR, ginger could be considered a harmless and possibly effective alternative option for women suffering from the symptoms of NVP. Large standardized randomized controlled trials are necessary to confirm the possible benefit of ginger as treatment for NVP.

## Competing interests

The authors declare that they have no competing interests.

## Authors’ contributions

The authors contributed the following: EV: Developed review protocol, selected RCTs, conducted data extraction, assessment of risk of bias in included RCTs, developed, edited and critically reviewed the manuscript. JV: Contributed to designing the review methodology, acted as third party arbitrator and critically reviewed the manuscript. NK: Contributed to designing the review and critically reviewed the manuscript. AM: Conducted the statistical analysis, interpretation of results and critically reviewed the manuscript. All authors read and approved the final manuscript.
